# Carbendazim residue in plant-based foods in China: Consecutive surveys from 2011 to 2020

**DOI:** 10.1016/j.ese.2023.100301

**Published:** 2023-07-11

**Authors:** Dou Wang, Guiling Yang, Xiao Yun, Ting Luo, Hao Guo, Liying Pan, Wei Du, Yanhua Wang, Qiang Wang, Pu Wang, Qinghua Zhang, Yun Li, Nan Lin

**Affiliations:** a State Key Laboratory for Managing Biotic and Chemical Threats to the Quality and Safety of Agro-products, Laboratory (Hangzhou) for Risk Assessment of Agricultural Products of Ministry of Agriculture, Institute of Agro-product Safety and Nutrition, Zhejiang Academy of Agricultural Sciences, Hangzhou, 310021, Zhejiang, China; b College of Urban and Environmental Sciences, Peking University, Beijing, 100871, China; c Yunnan Provincial Key Laboratory of Soil Carbon Sequestration and Pollution Control, Faculty of Environmental Science & Engineering, Kunming University of Science &Technology, Kunming, 650500, China; d Hubei Key Laboratory of Industrial Fume and Dust Pollution Control, School of Environment and Health, Jianghan University, Wuhan, 430056, China; e State Key Laboratory of Environmental Chemistry and Ecotoxicology, Research Center for Eco-Environmental Sciences, Chinese Academy of Sciences, Beijing, 100085, China; f Key Laboratory of Agro-Product Quality and Safety of Ministry of Agriculture, Institute of Quality Standards and Testing Technology for Agro-Products, Chinese Academy of Agricultural Sciences, Beijing, 100081, China; g Department of Environmental Health, School of Public Health, Shanghai Jiao Tong University, Shanghai, 200025, China

**Keywords:** Carbendazim, Plant-based foods, China, Spatial-temporal variations, Exposure and risk assessment

## Abstract

Carbendazim, a widely used fungicide in China, has been found to have reproductive toxicity and mutagenic effects. However, information on the spatial-temporal variations of carbendazim residues in food in China is limited. Here, we investigated the presence of carbendazim in China's plant-based foods from 2011 to 2020, evaluated the spatial-temporal characteristics, and assessed the associated exposure risks in the Chinese diet. The results revealed a high detection frequency of carbendazim in fruits (26.4%) and high concentrations in vegetables (∼110 mg kg^−1^), indicating widespread misuse of the fungicide. The acute risks of consuming certain vegetables and cereals exceeded the recommended limits by up to 12 and 5 times, respectively. Although there has been a decline in carbendazim residue levels in food since the implementation of the Chinese government's action plan for zero growth of pesticide use in 2015, some provinces still exhibited high levels of carbendazim in multiple food categories, which were positively correlated with annual pesticide application. We highlight that carbendazim contamination reflects the broader issue of pesticide use in China. It emphasizes the need for committed and targeted national policies to reduce carbendazim residues in food and suggests that such measures could also regulate the use of other pesticides, given that pesticide abuse in China is not limited to specific types. We call for the re-evaluation of maximum residue limits of carbendazim, particularly in highly consumed foods such as cereals.

## Highlights

• Spatial-temporal variations of carbendazim residue in China are portrayed.
• Committed national policies are beneficial for decreasing carbendazim residue.
• Carbendazim in provinces was positively associated with total pesticide usage.
• Carbendazim is an epitome to reflect the pesticide use in China.
• The exceedance of acute risks appealed for a redefining of carbendazim limits.

## Graphical abstract

**Figure:**
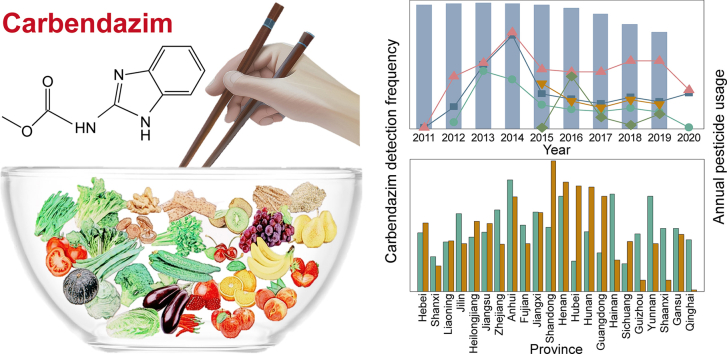

## 1 Introduction

Carbendazim is an efficacious and cost-effective systemic broad-spectrum fungicide against a wide variety of fungal diseases, such as mold, spot, mildew, scorch, rot, and blight. It serves a crucial function during the cultivation phase of crops and is also employed as a preservative in vegetables, fruits, cereals, and other agricultural products after harvest, and even in timber and paint [1]. Carbendazim has a hundred days of half-life in soil and water and thus remains persistent in the environment [2]. Moreover, it has also been found to be stable after processing by pasteurization, cooking, brewing, and sterilization in matrices of plant origin [3,4]. Although carbendazim has a relatively short half-life of only a few days in plants [5], its residues are frequently detected in fruits and vegetables, which may be attributed to its frequent and extensive application.

Carbendazim has been designated as a Group C class of Possible Human Carcinogen by the United States Environmental Protection Agency (US EPA) due to its toxic properties and potential for bioaccumulation [6,7]. To date, carbendazim application in human food has been prohibited in many countries and regions, including the European Union (EU), the US, and Canada, primarily due to its reproductive toxicity (irreversible male infertility) and mutagenic effects (e.g., embryotoxicity and birth defects) [[8], [9], [10], [11]]. Previous studies also suggest that sub-chronic carbendazim exposure via oral ingestion can induce hepatic lipid metabolism disorder and gut microbiota dysbiosis in adult zebrafish and mice [12,13]. Residue concentrations (∼34.8 mg kg^−1^ in vegetables) that could threaten human health have also been detected in agricultural commodities in both China and other countries [14,15], raising concerns about food safety problems. Additionally, carbendazim is not only intentionally produced as a pesticide but is also a main metabolite of thiophanate-methyl and benomyl fungicides [14]. Thiophanate-methyl and carbendazim exhibit similar toxicological effects, which warrants their consideration in a combined risk assessment. Notably, carbendazim has demonstrated significantly higher toxicity than thiophanate-methyl, but even so, carbendazim products are still widely utilized and even abused in many countries, particularly in developing countries with large agricultural production.

The increasing globalization of the world's economy has also brought more attention to the issue of food safety caused by pesticide residues. The lack of standards or regulations in food production and manufacturing in some countries occasionally triggers international import and export trade conflicts. A notable instance involves carbendazim contamination in orange juice, which led the United States Food and Drug Administration (US FDA) to enforce more rigorous inspections and prerequisites on orange juice imports from Brazil [16], with a subsequent extension of these measures to additional imported food products [17]. Nevertheless, pesticides have been deemed to contribute to the yield and quality of agricultural production, particularly in the context of climate change's substantial impact on agriculture. Consequently, an increasing number of countries and regions have initiated pesticide residue monitoring programs and established more stringent maximum residue limits (MRLs), such as the US [17], EU countries [18], and South Korea [19], all of which have included carbendazim in their target lists.

China is a large agricultural country and contributes the largest yield of cereals, vegetables, and fruits in the world, along with the largest production and use of pesticides, including carbendazim. In 2017, the estimated global annual production of carbendazim was about 40,000 tons, and most were produced in China, where 25% of the output was domestically consumed. Brazil, India, and Argentina are the main importers of carbendazim [20], and carbendazim still remains a prevalent fungicide elsewhere in the world market. In 2015, China's Ministry of Agriculture issued an action plan for zero growth of pesticide use by 2020 [21], proposing to promote green prevention, coordinated control, and scientific pesticide use and to carry out zero increase in pesticide use. In 2016, 929 single and mixed products of carbendazim were registered for agricultural production, including for crops, fruits, vegetables and other economic crops in China. By 2022, the number decreased to 578, and 303 thiophanate-methyl relevant products were registered [22]. However, due to the lack of alternative products, carbendazim may not be banned in agricultural production in a short term in China. Several previous studies have found carbendazim residue in specific foods in China [23], but the systematic and comprehensive picture is still unknown, which hinders the establishment of precise control measures.

Importantly, the Chinese government has been reviewing the risks posed by carbendazim from agricultural production in China since 2020. Based on this comprehensive and target risk assessment project, we identified carbendazim residue in 66 agricultural commodities in five categories of plant-based foods (vegetables, fruits, mushrooms, cereals, and tea) from a total of 31 provincial-level administrative divisions (“provinces”) in China for 10 consecutive years (2011–2020). After this, we implemented probabilistic simulations for dietary exposure to carbendazim to assess the chronic and acute health risks in more realistic consumer scenarios. Therefore, the objectives of this study are (1) to investigate carbendazim in a wide range of agricultural products, then (2) to characterize the spatial-temporal variability of carbendazim residue in China over a decade, (3) to explore the influence of relevant national policies and activities on carbendazim residue, and finally, (4) to assess human exposure regarding the Chinese diet and to evaluate the possible protection offered by MRLs. We expect the results to guide more detailed management and control measures for carbendazim use in China.

## 2 Materials and methods

### 2.1 Sample collection

A comprehensive field survey was carried out across 31 provinces in China, spanning seven geographic regions from 2011 to 2020 (Fig. S1). The study encompassed 117,289 samples, comprising 74,029 vegetables, 24,607 fruits, 17,275 mushrooms, 908 cereals, and 470 teas (Table S1). These samples encompassed the primary constituents of the Chinese diet, including 37 types of vegetables, 17 types of fruits, 9 types of mushrooms, 2 types of cereals, and Chinese tea. The sample collection was based on annual pesticide monitoring programs for agricultural products in China during ten years, and all were carried out by trained and accredited personnels from several authoritative laboratories attached to research institutes and universities throughout China. Samples were randomly taken from two sources: the production process, including farms and production bases, and the consumption process, including supermarkets and wholesale markets. The samples from the productive process were collected when the products were ripe or ready for sale. Nonseasonal crops were generally collected quarterly, and seasonal crops were obtained during their peak selling periods.

Vegetable, fruit, and mushroom collection followed the guidelines on sampling for pesticide residue analysis of NY/T 789–2004 [24], and cereal and tea sampling were performed following the method described in NY/T 5344.2–2006 [25] and GB/T 8302–2013 [26], respectively. Then, 2–3 kg of fresh samples was wrapped in a labeled plastic bag and transported to the laboratory within 24 h, avoiding deterioration, damage, water loss, and contamination. A 250–500 g aliquot was then acquired by quartering and transferred into a polyethylene container after cutting into small pieces or homogenization. All processed samples were stored at −20 °C until analysis.

### 2.2 Laboratory analysis

***Chemicals and reagents.*** HPLC grade *n*-hexane, acetonitrile, and toluene were purchased from Thermo Fisher (Fair Lawn, NJ, USA). The carbendazim standard was from the Agro-Environment Protection Institute, the Ministry of Agriculture and Rural Affairs of China (Beijing, China). The standard solution was prepared by diluting the stocks with acetonitrile, and purification materials, such as C_18_ (50 μm), PSA (40–60 μm), NaCl, and anhydrous MgSO_4_ were of guaranteed grade (purity >99.8%) from Agela Technologies Inc (Torrance, USA). Sep-Pak NH_2_ cartridges were obtained from Waters (Dublin, Ireland). Finally, syringe filters (0.22 μm; Tengda, Tianjin, China) were used to filter the concentrated extracts.

***Sample preparation.*** The sample pretreatment followed the China National Standard using a modified QuEChERS procedure [27]. Briefly, an accurately weighed sample (10.0 g for vegetables, fruits, and mushrooms; 5.0 g for cereals; 2.0 g for tea) was homogenized with 10.0 mL of acetonitrile for 3 min. Then, 1.5 g of NaCl and 6 g of anhydrous MgSO_4_ were added. These mixtures were vortexed for 1 min and centrifuged at 7000 r min^−1^ for 3 min. Next, 1.5 mL of acetonitrile extract was transferred to a 2 mL centrifuge tube containing PSA (50 mg), C_18_ (50 mg), and MgSO_4_ (150 mg), and the centrifuge tube was vortexed for 1 min and centrifuged at 7000 r min^−1^ for 1 min. Finally, the supernatant was filtered through a filter (0.22 μm) for instrumental analysis.

***Instrumental analysis.*** The Carbendazim measured in this study included carbendazim, thiophanate-methyl, and benomyl. The carbendazim was analyzed using triple quadrupole liquid chromatography-tandem mass spectrometry (LC-MS 8050, Shimadzu, Kyoto, Japan) with an ACE C_18_ column (2.1 × 100 mm, 1.7 μm; Phenomenex, Torrance, CA, USA). The mobile phase was composed of 5 mmol L^−1^ ammonium formate aqueous solution (A) and methanol (B) at a flow rate of 0.3 mL min^−1^. Solvent B was initially set at 5% and increased to 40% in 1 min, ramped to 80% over 2 min, then to 95% over 2 min, held for 2 min, then returned to 5% in 0.1 min and held for 2 min. Multi-reaction monitoring was conducted using an electrospray voltage of 4.0 KV, and *m/z* 192.05→*m/z* 160.05 (collision energy 17 eV) and *m/z* 192.05→*m/z* 132.05 (collision energy 30 eV) were employed for quantification and identification, respectively. Electrospray ionization in positive ion mode was used with a capillary temperature of 300 °C, and the desolvation line and heat block temperatures were set to 250 °C and 400 °C, respectively. The chromatograms of the carbendazim are shown in Fig. S2.

### 2.3 Quality assurance (QA) and quality control (QC)

Calibration curves were obtained by testing matrix-matched calibration standards ranging from 1 to 250 μg L^−1^, and the linearity of coefficients of determination (R^2^) was all higher than 0.993. The limit of detection (LOD) of this method was defined as three times the signal-to-noise of the peak determined based on the matrix blank at low concentration. The LODs were 0.00012–0.01 mg kg^−1^ in vegetables, 0.0001–0.07 mg kg^−1^ in fruits, 0.001 mg kg^−1^ in mushrooms, 0.01–0.05 mg kg^−1^ in cereals, and 0.001 mg kg^−1^ in tea, and the limit of quantification (LOQ) of the method was 0.01 mg kg^−1^. A total of 25–30 samples were taken as a batch with a blank sample and matrix-matched calibration standards, and solvent blanks and calibration curves were analyzed before the operation of each batch. No carbendazim was detected in the blanks. The samples were quantified by matrix standard. Finally, the recoveries of four spiked concentrations (0.01, 0.1, 1, and 10 mg kg^−1^) ranged from 81.2% to 118.3%, and the precision calculated as relative standard deviation ranged from 1.8% to 10.2%.

### 2.4 Exposure and risk assessment

We assessed both chronic and acute risks of dietary exposure to carbendazim from consuming four categories of food, vegetables, fruits, cereals, and potatoes. Potatoes were separated from vegetables based on existing consumption information (Table S2) from the Fifth China Total Diet Study (TDS) [28]. Five populations, including children (2–7 years), adolescent males (8–19 years), adolescent females (8–19 years), adult males (>20 years), and adult females (>20 years), were considered for the assessment. Life expectancy was assumed to be 80 years, and the acceptable daily intake (ADI) and acute reference dose (ARfD), both 0.02 mg kg^−1^ day^−1^, developed by the European Food Safety Authority (EFSA), were used for chronic and acute risk assessment. Detailed information is presented in SI. Finally, Monte Carlo simulations [29] were performed in chronic and acute exposure assessments to provide more useful information on the risks and enable us to describe the population using probability [[30], [31], [32]].

### 2.5 Data analysis

The detection frequency of residue concentrations ≥ LOQ (0.01 mg kg^−1^), set as DF_≥0.01_, was chosen so that our subsequent analysis would be consistent with and comparable to international guidelines and studies. Since the detection frequency was generally low (<50%, Table 1), median concentrations among samples with residue ≥0.01 mg kg^−1^ (median_≥0.01_) were also considered. The original DF and DF_≥0.01_ presented a significant correlation (*p* < 0.05, Spearman's correlation analysis), indicating that discussion using DF_≥0.01_ could inform the characteristics of the original DF. The association between DF_≥0.01_ and median_≥0.01_ also presented a significant correlation (*p* < 0.05, Spearman's correlation analysis, Fig. S3). Annual pesticide usage from 2011 to 2019 and annual agricultural production (including vegetables, fruits, and cereals) from 2011 to 2018 in 22 provinces were obtained from http://www.stats.gov.cn/tjsj/.

**Table 1:** Sample size, the detection frequency of carbendazim residue ≥0.01 mg kg^−1^ (DF_≥0.01_), maximum residue limits (MRLs) set in national standards or ministry guidelines, over-limit ratio (OLR), and detailed carbendazim residue values including median concentrations among samples with residue ≥0.01 mg kg^−1^ (median_≥0.01_) of the 66 types of target foods.

Category	Name	Sample size	DF_≥0.01_	MRL (mg kg^−1^)	OLR (%)	Concentration (mg kg^−1^)^a^		
						Median	Median_≥0.01_	Max
**Vegetable**	Tomato	4250	13.7%	3	0.00%	ND	0.03	2.46
**(*N*** = **37)**	Leguminous vegetables	4305	17.1%	0.5	0.51%	ND	0.05	2.13
	Eggplant	3965	6.10%	3	0.00%	ND	0.03	0.80
	Balsam pear	1605	9.20%	2^b^	0.00%	ND	0.04	1.20
	Pumpkin	136	5.10%	2^b^	0.00%	ND	0.05	0.28
	Summer squash	2358	2.50%	0.5	0.00%	ND	0.02	0.45
	Sponge gourd	223	6.30%	2^b^	0.00%	ND	0.03	0.07
	Chinese wax gourd	124	3.20%	2^b^	0.00%	ND	0.02	0.05
	Cucumber	3967	15.7%	2	0.00%	ND	0.04	0.78
	Okra	12	0.00%	0.5^b^	0.00%	ND	0.00	0.00
	Hot pepper	4465	9.90%	2	0.02%	ND	0.04	2.29
	Chinese Cabbage	7423	6.30%	0.5^b^	0.86%	ND	0.07	6.90
	Spinach	1864	7.20%	0.5^b^	1.61%	ND	0.11	110
	Amaranth	318	10.7%	0.5^b^	0.94%	ND	0.09	15.0
	Bok choy	957	10.1%	0.5^b^	1.88%	ND	0.08	24.0
	Water spinach	1387	7.00%	0.2^b^	1.01%	ND	0.05	3.61
	Chinese chive	1984	10.3%	2	0.60%	ND	0.08	15.0
	Cabbage flowering stalk	1370	6.60%	0.5^b^	0.73%	ND	0.06	2.73
	Coriander leaf	291	6.90%	0.5^b^	0.00%	ND	0.03	0.13
	Endive lettuce	181	24.9%	5^b^	6.63%	ND	0.09	22.0
	Romaine lettuce	154	20.1%	5^b^	2.60%	ND	0.11	19.0
	Vinespinach	153	16.3%	5^b^	0.00%	ND	0.05	2.60
	Celery stem	3007	18.4%	0.5^b^	3.29%	ND	0.08	35.0
	Cabbage	3294	1.20%	0.5	0.03%	ND	0.02	21.0
	Common spiderflower	3919	1.60%	0.5^b^	0.03%	ND	0.03	3.10
	Leaf mustard	149	8.70%	0.5^b^	1.34%	ND	0.04	0.65
	Crown daisy	203	8.40%	5^b^	0.00%	ND	0.05	2.09
	Yam	1414	13.4%	0.2	3.25%	ND	0.08	11.1
	Potato	2078	0.60%	0.2^b^	0.00%	ND	0.03	0.12
	Lotus root	992	2.20%	0.2	0.10%	ND	0.06	1.80
	Radish & Carrot^c^	4520	0.80%	0.2^d^	0.00%	ND	0.03	0.19
	Lettuce stem	3825	11.2%	5	0.26%	ND	0.12	21.0
	Manchurian wild rice	2079	3.10%	0.2^b^	0.10%	ND	0.02	2.40
	Water chestnut	475	1.50%	-	0.00%	ND	0.02	0.21
	Arrowhead	271	2.60%	-	0.00%	ND	0.02	0.10
	Asparagus	35	0.00%	0.5	0.00%	ND	0.00	0.01
	Allium vegetables	6276	2.80%	2^b^	0.03%	ND	0.06	4.27
	**Subtotal**	**74029**	**7.70%**	**-**	**0.48%**	**ND**	**0.05**	**110**
**Fruit**	Watermelon	3500	6.00%	2	0.00%	ND	0.03	0.40
**(*N*** = **17)**	Casaba	73	15.1%	-	0.00%	ND	0.02	0.30
	Apple	2794	48.5%	5	0.00%	ND	0.04	1.54
	Pear	1767	19.9%	3	0.00%	ND	0.04	2.90
	Plum	72	16.7%	0.5	0.00%	ND	0.04	0.09
	Peach	2666	47.1%	2	0.23%	ND	0.06	8.39
	Chinese date	1410	41.9%	0.5	0.43%	ND	0.05	1.10
	Pomegranate	115	53.9%	0.5^b^	13.04%	0.02	0.17	6.05
	False sour cherry	347	35.7%	0.5	0.00%	0.001	0.04	0.34
	Bayberry	800	16.3%	1	0.25%	ND	0.00	2.32
	Loquat	58	5.20%	3	0.00%	ND	0.10	0.30
	Lychee	110	5.50%	0.5	0.00%	ND	0.01	0.02
	Banana	1598	33.8%	2	0.00%	ND	0.05	1.67
	Citrus	2634	16.7%	5	0.00%	ND	0.03	1.20
	Grape	3248	19.2%	3	0.00%	ND	0.05	1.60
	Strawberry	2635	20.4%	0.5	0.80%	ND	0.05	2.64
	Chinese kiwi fruit	780	32.1%	5	0.00%	0.0006	0.07	2.59
	**Subtotal**	**24607**	**26.4%**	**-**	**0.20%**	**ND**	**0.05**	**8.39**
**Mushroom**	Shitake mushroom	5025	13.4%	3^b^	0.02%	ND	0.03	4.47
**(*N*** = **9)**	Button mushroom	1992	34.2%	3^b^	0.10%	ND	0.10	4.85
	Oyster mushroom	4244	2.90%	3^b^	0.00%	ND	0.03	0.63
	Columnar agroc	762	2.40%	3^b^	0.00%	ND	0.04	1.20
	Gold needle mushroom	3741	9.50%	3^b^	0.00%	ND	0.05	2.13
	King oyster mushroom	471	1.30%	3^b^	0.00%	ND	0.03	0.08
	Straw mushroom	363	13.8%	3^b^	0.00%	ND	0.04	0.41
	Wood ear fungus	629	6.70%	3^b^	0.48%	ND	0.13	35.4
	Silver ear fungus	48	2.10%	3^b^	0.00%	ND	0.01	0.01
	**Subtotal**	**17275**	**11.3%**	**-**	**0.03%**	**ND**	**0.05**	**35.4**
**Cereal**	Rice	498	2.20%	2	0.20%	ND	0.02	5.20
**(*N*** = **2)**	Wheat	410	13.2%	0.5	0.73%	ND	0.03	0.94
	**Subtotal**	**908**	**7.20%**	**-**	**0.44%**	**ND**	0.03	**5.20**
**Tea (*N*** = **1)**	**Tea**	**470**	**0.90%**	**5**	**0.00%**	**ND**	**0.02**	**0.04**
**Total**		**117289**	**12.2%**	**-**	**0.35%**	**ND**	**0.05**	**110**

a ND means none detected, set as 1/2 of LOD.

b MRLs are from the guidelines of the Ministry of Agriculture and Rural Affairs of the People's Republic of China.

c Radishes and carrots were mixed when recording the data.

d MRL (0.2 mg kg^−1^) is for carrot (Radish has no MRL).

Differences between food categories were evaluated using Mann-Whitney U tests (two samples) and Kruskal-Wallis tests (three or more samples), and correlations were tested using Spearman's correlation analysis. A principal component analysis (PCA) was also performed to identify potential sources. All analysis was carried out using SPSS (IBM, Inc., Chicago, Illinois, USA) and R (R Foundation, Vienna, Austria), and all the statistical tests were two-sided with a type-I error rate of 0.05.

The Monte Carlo simulations were performed for three main parameters: daily food consumption, body weight, and carbendazim concentration. Daily food consumption and body weight were simulated with a variable coefficient of 10% based on the mean value from the Fifth China TDS (Table S2) and were 10,000 times using MATLAB 2021a (The MathWorks, Inc., Natick, Massachusetts, USA).

## 3 Results

### 3.1 Carbendazim residue levels

Carbendazim was higher than 0.01 mg kg^−1^ in 14,253 of the 117,289 total food samples, indicating a DF_≥0.01_ of 12.2% (Table 1). 7.70% of vegetables, 26.4% of fruits, 11.3% of mushrooms, 7.20% of cereals, and 0.90% of tea had carbendazim ≥0.01 mg kg^−1^. Among the 66 varieties of plant-based foods, only two (okra and asparagus) showed no carbendazim residue, which may be related to the small sample sizes (*N* = 12 and 35, respectively). Fruits generally had higher DF_≥0.01_ (26.4%) than the other four categories (*p* < 0.001), and among all fruits, four (pomegranate, apple, peach, and Chinese date) had DF_≥0.01_ higher than 40%; 10 kinds ranged from 15% to 36%; and only three kinds (loquat, lychee, and watermelon) had DF_≥0.01_ less than 10%. Among the vegetables, the leafy vegetables, such as endive lettuce, romaine lettuce, vinespinach, and celery, had the highest DF_≥0.01_, from 16% to 25%. For mushrooms, button mushrooms had a DF_≥0.01_ of 34.2%, which was much higher than any other type of mushrooms, and for cereals, wheat had a higher DF_≥0.01_ than rice. All these findings reveal the extensive use of carbendazim in the agricultural industry in China, especially in fruits.

The median_≥0.01_ residue levels among vegetables, fruits, and mushrooms were similar, at about 0.05 mg kg^−1^ (Table 1 and Fig. S3). Vegetables with higher DF_≥0.01_ also had a higher median_≥0.01_ as well. For example, endive lettuce, romaine lettuce, and celery had median_≥0.01_ close to 0.10 mg kg^−1^, reinforcing the result of more severe contamination in leafy vegetables. Regarding fruits, the highest median_≥0.01_ was also found in pomegranates, up to 0.17 mg kg^−1^, but all other fruits had median_≥0.01_ lower than 0.10 mg kg^−1^. For mushrooms, the highest median_≥0.01_ was observed in wood ear fungus, but button mushrooms also had a relatively high median_≥0.01_. Extremely high residue levels were also often detected in vegetable samples (Table 1). The highest carbendazim residue concentrations in ten kinds of vegetables were over 10 mg kg^−1^ and up to 110 mg kg^−1^ (spinach). In addition, the highest residue levels in 17 types of fruits were lower than 10 mg kg^−1^. Mushrooms showed relatively low levels of carbendazim residue, except wood ear fungus, with the highest level of 35.4 mg kg^−1^. Therefore, carbendazim exposure is more likely to occur when eating fruit, but vegetables are the main foods that contain high levels of carbendazim.

### 3.2 Temporal and spatial variations

**Fig. 1:**
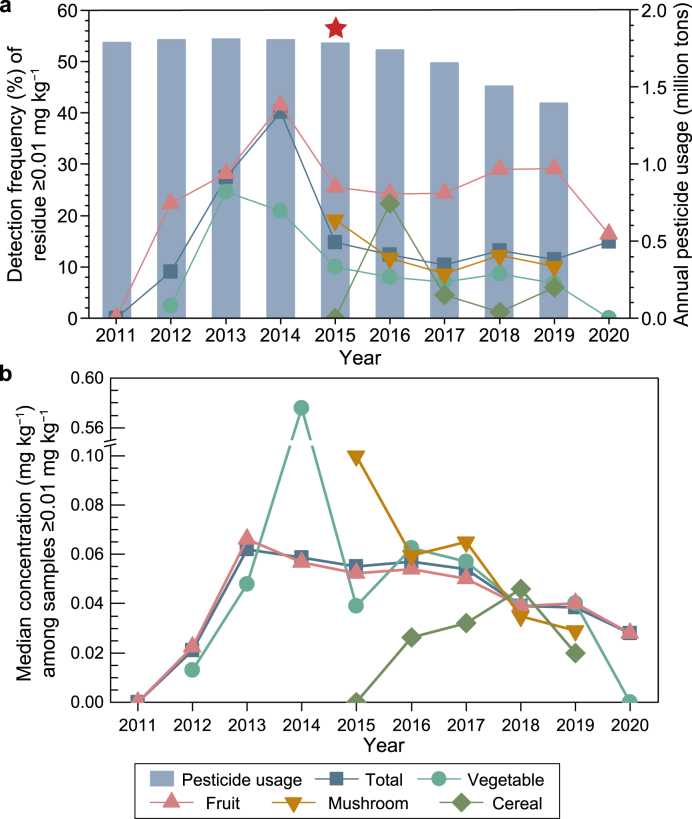
**a**, Detection frequency of carbendazim over 0.01 mg kg^−1^ of total food samples, vegetables, fruits, mushrooms, and cereals along with annual pesticide usage in China from 2011 to 2020. The annual pesticide usage in 2020 is not yet available. The red star refers to the year when China's Ministry of Agriculture issued the action plan for zero growth of pesticide use by 2020. **b**, Median concentration among samples with carbendazim over 0.01 mg kg^−1^ in total food samples, vegetables, fruits, mushrooms, and cereals from 2011 to 2020.

The temporal DF_≥0.01_ and median_≥0.01_ of carbendazim for the total and each category except tea are available in Fig. 1. The DF_≥0.01_ of the total, vegetables, and fruits increased from 2011 to 2014, then decreased to a steady phase starting in 2015 (Fig. 1a). Mushrooms were sampled from 2015 to 2019, which also presented a decrease in 2015 (21.8%) and then a steady phase beginning in 2016 (12.0%). In terms of cereal, DF_≥0.01_ decreased dramatically since 2017 (9.2%). As for total food samples, the low DF_≥0.01_ in the early years, especially in 2011, may relate to the small sample size (70 samples total in 2011), but median_≥0.01_ began to decline in 2014, then kept a relatively stable state from 2015 to 2016, and dropped significantly from 2017 to 2020 (Fig. 1b). Overall, carbendazim residue in all categories of foods began the decline around 2015 and maintained a low detection frequency along with a decreasing residue concentration in the following years.

We also characterized the spatial variations of carbendazim DF_≥0.01_, see Fig. S4, to analyze the provincial differences by total plant-based foods and each category except cereals and tea, which were sampled in only a few provinces. All 31 provinces had DF_≥0.01_ of total foods over 5%, and most (22 out of 31) were over 10%. There was no significant agglomeration in geographical distribution. However, in several provinces, if DF_≥0.01_ of one food category was high, the DFs of the other categories tended not to be low. Chongqing (CQ) and Xizang (XZ), both located in southwest China (Fig. S1), had the highest DF_≥0.01_ for vegetables (16.8% and 15.5%), fruits (50.3% and 29.4%), and mushrooms (10.3% and 19.1%), and Henan (HEN) had the highest DFs (23.1%) of cereals (only wheat) and relatively high DFs of fruits (34.3%) and mushrooms (15.9%). These results further demonstrate the widespread use of carbendazim in agricultural products in China and the food contamination across the board caused by improper regulation in certain provinces.

### 3.3 Exposure and risk assessment

**Fig. 2:**
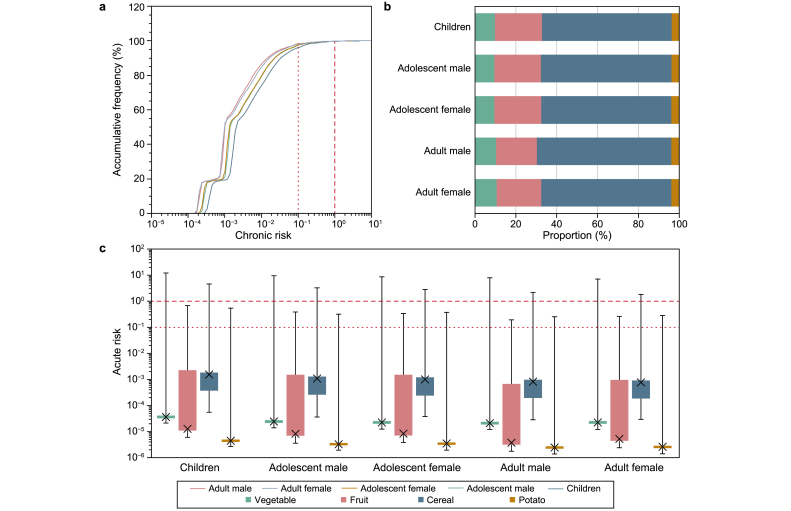
**a**, Accumulative frequency of chronic risks of dietary exposure to carbendazim from the combined intake of four food categories (vegetables, fruits, cereals, and potatoes) in five populations. The two red dashed lines represent typical protective reference risk levels, 0.1 and 1. **b**, Mean contributions of four food categories to chronic risks in five populations. **c**, Boxplots of acute risks of dietary exposure to carbendazim from each food category (vegetables, fruits, cereals, and potatoes) in five populations. The two red dashed lines represent typical protective reference risk levels, 0.1 and 1.

For acute exposures to different foods and chronic exposure to a combined diet, there was a gradual decrease from children to adolescents, then to adults (Table S3), mainly due to increasing body weight with age. Most chronic risks (CRs) were much lower than 1 and even 0.1. Less than 0.5% of people had chronic risks over 1, and less than 5% had chronic risks over 0.1 (Fig. 2a), indicating that chronic exposure risks of carbendazim may not require vigilance. In terms of different food contributions (based on mean exposure), cereals made up the largest proportion of total dietary exposure to carbendazim due to their large consumption of the Chinese diet (Fig. 2b).

Regarding acute risks (ARs), the highest levels derived from food intake had a 97.5% residue concentration, far exceeding the ARfD when consuming vegetables and cereals across all five populations (Fig. 2c). The high residues measured in vegetables (e.g., 110 mg kg^−1^ of spinach and 35 mg kg^−1^ of celery, Table 1) and high daily intake of vegetables (e.g., averaging 0.19 kg day^−1^ for children and 0.44 kg day^−1^ for adult males) contributed to the highest ARs (7.1–12.1). Although the highest carbendazim concentration in cereals was merely 5.2 mg kg^−1^ found in rice, the high daily intake of cereals in the Chinese diet (e.g., averaging 0.22 kg day^−1^ for children and 0.45 kg day^−1^ for adult males) resulted in the ARs of cereal consumption exceeding the ARfD of 0.02 mg kg^−1^ day^−1^, a factor of up to 4.6 for children.

## 4 Discussion

### 4.1 Carbendazim residue levels in literature

Carbendazim has been detected in various foods in many countries, even where its use in agricultural products for human consumption has been banned (Table S4). Vegetables and fruits have been the main categories of food investigated in previous studies, and the DF of vegetables in China has been found to be nearly in the middle and even at low levels, comparable to Pakistan, Turkey, and Cameroon [15,[33], [34], [35], [36]], but significantly lower than Columbia, Nepal, and Kenya [[37], [38], [39]]. Even for specific vegetables, such as tomatoes, the DF_≥0.01_ in our study was 13.7%, lower than 19.5% in Colombia in 2011, although the highest concentration was 2.50 mg kg^−1^, higher than the 0.74 mg kg^−1^ in Colombia [39]. A previous study conducted in China also supports these findings [14].

Pesticide monitoring data in the EU reported a relatively higher amount of carbendazim in vegetables imported from several developing countries, including Uganda, India, Pakistan, and Kenya [40]. In terms of fruits, the DFs in China were about 3–9 times higher than those in other countries. Peaches and nectarines in Spain in 2002 had a DF of 34% (LOD = 0.02 mg kg^−1^), equating to 40.0% in the present study if the LOD was set using our method. The highest residue concentration of peaches in our study was 8.4 mg kg^−1^, almost ten times that in Spain (0.9 mg kg^−1^) [41]. Another work reported that peaches in China had a DF (LOD = 0.0004 mg kg^−1^) of 60.6% with the highest concentration of 3.40 mg kg^−1^ [42]. Additionally, compared to developed countries, such as the US [17], China had more foods with higher carbendazim levels.

In general, the carbendazim DF of vegetables in China was low, but the DF of fruits was quite high. Moreover, the high concentrations were usually several times higher than those in other countries. As mentioned above, previous studies have usually focused on one food category in a limited period, for example, only vegetables during 2014–2016 [14] or fruits in 2010–2012 [15]. We included a wide range of samples over a decade, presenting a more comprehensive picture of carbendazim residue in almost all relevant foods.

### 4.2 Different categories of agricultural products

The carbendazim DF of fruits in China was higher than those in other countries but also significantly higher than other food categories in China. Remote sales of fruits (even exports) account for a much larger proportion than local sales. Fruits are not the priority category in the Vegetable Basket Project in China, which has been implemented for over 30 years to alleviate the shortage of agricultural products supplies [43]. Many local governments are paying more and more attention to maintaining their self-sufficiency rates (supplying themselves from local production) for mainly vegetables, meats, and eggs [44,45]. Accordingly, the fruit has a longer period from being picked to being consumed than other foods in China. During the post-harvest period, fungal diseases (e.g., apple penicillium disease or gray mold on strawberries) are the dominant cause of fruit spoilage [46], which has led to a large increase in the use of antifungal agents compared to other categories of food. For example, according to China Pesticide Registration Data [22], thiophanate-methyl can be used in citrus fruit immersion.

Relatively higher DFs and concentrations were observed in leafy vegetables than other vegetables, in agreement with previous studies [[47], [48], [49]]. Leafy vegetables have a larger surface area compared to their weight, leading to higher area-to-mass ratios and higher rates of detection of pesticides [48]. Moreover, leafy vegetables usually have a short growth cycle but a high incidence of disease, which gives rise to a short interval between pesticide applications [50]. Insufficient time for pesticide degradation before harvest may therefore be another cause for the higher DFs and concentrations of carbendazim residue in leafy vegetables.

Regarding the higher DF and concentration of carbendazim in wheat than in rice, it may be due to the need to control Fusarium head blight, a worldwide devastating disease of wheat [51,52]. Furthermore, wheat has also been found to have a higher residue of other pesticides. For example, chlorinated pesticide residues (chlorpyrifos methyl) were present in most of the wheat or wheat flour samples in Kuwait from 1995 to 1996 [53], and among the four grains, wheat had the highest detection frequency (29.0%) and concentration of pesticide residue in Kazakhstan [54]. Multiple pesticide residues were also detected in half of wheat flour samples from Beijing, China, and carbendazim was the most frequently detected pesticide, with a detection rate of 98.6% and 94.3% in 2019 and 2020, respectively [55].

Our PCA results for samples with carbendazim higher than 0.01 mg kg^−1^ identified three major factors similar to the food category (Fig. S5). Factor 1, accounting for 30% of the variance, included mainly vegetables, such as leguminous vegetables, balsam pear, hot peppers, and celery, mainly fruits of the vegetable plants. Two kinds of pome and stone fruits, apples and peaches, had loadings of 83% and 80%, respectively, in Factor 1. Factor 2 accounted for 24% of the variance and included mainly fruits, such as bananas, strawberries, citrus. Cucumbers and shiitake mushrooms also had abundant loadings, as high as 93% and 87%, respectively. Factor 3, accounting for 20% of the variance, only included gold needle mushrooms. Other factors accounted for much less variance and are not shown in Fig. S5.

### 4.3 Temporal and spatial variations

The DFs and concentrations of all categories of foods began to decrease around 2015, when China's Ministry of Agriculture issued the action plan for zero growth of pesticide use, as mentioned above [21]. Since then, pesticide usage in China has declined for five consecutive years (Fig. 1a), along with chemical pesticide production [56]. The logistics and transportation industry, including cold-chain transportation, showed a significant increase around 2015 as well. This may also play an important role in promoting the sale of agricultural products, helping to alleviate carbendazim use in preventing agricultural products from spoiling. In our comparison of MRLs in China over the years, we did not find stricter changes, except for the addition of some foods to the list (Table S1). Therefore, the benefits of specific national policies can be directly reflected in pesticide residues in agricultural products. Crucially, even though pesticide residues have been reduced considerably since 2015, the Chinese government continues to promote the reduction of pesticide use and is actively taking further measures to ensure science-based and “green” agricultural production, along with food safety and health measures as well.

**Fig. 3:**
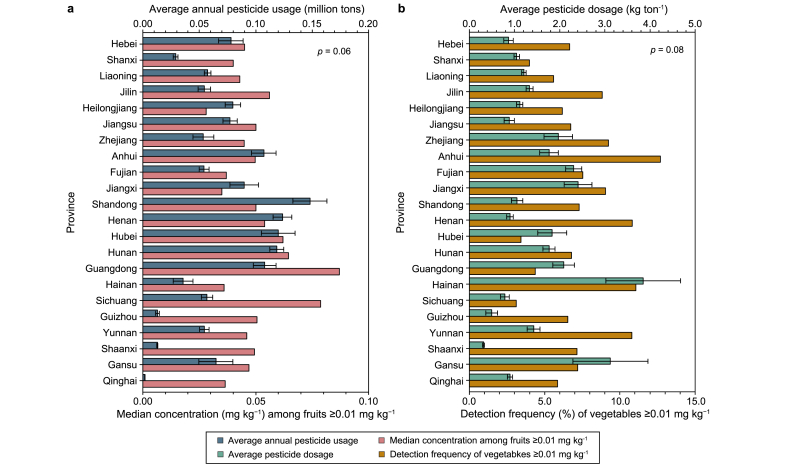
**a**, Average annual pesticide usage from 2011 to 2019 and median concentrations among fruits with carbendazim over 0.01 mg kg^−1^ in 22 provinces. **b**, Average pesticide dosage for vegetables, fruits, and cereals from 2011 to 2018 and detection frequency of vegetables with carbendazim over 0.01 mg kg^−1^ in 22 provinces. Both showed slight positive correlations via Spearman's correlation analysis (*p* = 0.06 and 0.08).

The high DFs and concentrations of carbendazim found in several provinces usually encompass multiple categories of agricultural products rather than being concentrated in one single category. A slightly positive correlation (*p* = 0.06) was found for annual pesticide usage and median_≥0.01_ among fruits in provinces (Fig. 3a). Shandong (SD) and Henan (HEN), two of the largest agricultural-producing provinces in China, applied more pesticides annually than others as well. The mean annual pesticide usage in SD and HEN from 2011 to 2019 was 0.15 ± 0.02 and 0.12 ± 0.01 million tons, respectively [56], higher than most provinces. The DF_≥0.01_ of fruits in SD and HEN were both higher than 30% and the median_≥0.01_ were both higher than 0.05 mg kg^−1^. We also calculated the ratio of annual pesticide usage to annual agricultural production (including vegetables, fruits, and cereals) in order to assess the average pesticide dosage (kg ton^−1^) in each province from 2011 to 2018. Pesticide dosage and DF_≥0.01_ among vegetables presented a slight positive correlation (*p* = 0.08, Fig. 3b). Hainan (HN) province had the highest average pesticide dosage (3.85 ± 0.83 kg ton^−1^), and as discussed above, had high carbendazim DF_≥0.01_ for vegetables (11.1%), fruits (50.3%), and mushrooms (19.1%) (Fig. S4).

Jiangxi (JX) and Hunan (HUN) provinces had relatively high levels of both annual pesticide usage (0.09 ± 0.001 and 0.12 ± 0.006 million tons, respectively) and pesticide dosage (2.41 ± 0.31 and 1.76 ± 0.13 kg ton^−1^, respectively) that may be relevant to the high carbendazim residues in multiple categories of foods. From the associations with total pesticide usage and dosage, we speculate that other pesticides may present a similar spatial distribution as the mixed application of pesticides is highly prevalent in China, and thus their overuse is likely not limited to a single pesticide. Due to the lack of annual pesticide usage and agricultural production data in Xizang and Chongqing, proposing a reasonable explanation for the highest-observed carbendazim DFs in these two provinces is difficult. The limited variety of food samples may also have influenced the results. For instance, the DF_≥0.01_ of fruits in 2015 in Chongqing was 72.9%, with sampling restricted to pomegranates and Chinese kiwi fruit (both of which demonstrated high carbendazim DF_≥0.01_). The fruit DF_≥0.01_ in other years in Chongqing that included more kinds of fruits remained relatively high (43.6–49.0%).

### 4.4 Exposure and risk assessment

As discussed above, the median levels of chronic health risk were far below 1 and even 0.1, suggesting a neglectable level. For acute risk, however, the highest or 97.5th percentile should be noted since it is set to assess the potential acute health risk derived from a large consumption of certain food containing a high pesticide residue during one meal or one day in an extreme case [57]. Here, we thus simulated the distributions of food intake and body weight among the Chinese population using Monte Carlo methods and the residue concentrations in our study. The upper residue intake per body weight amounted to the 99.9998th percentile from the 97.5th percentile of food consumption, the 97.5th percentile of concentration, and the 2.5th percentile of body weight [1 – (1–0.975) × (1–0.975) × 0.025] [57]. All five populations showed an extremely high acute health risk from eating vegetables and cereals (∼12.1 and ∼4.6, respectively). If processing factors are included in the assessment (Tier four), low to 0.1 [58,59], the acute risks would still reach or even exceed 1. Even so, the assessment in this study may be an underestimate. Although carbendazim residues are commonly detected in plant-based foods, recent studies have also reported their presence in meat products [60,61], which was not considered in our investigation. Along with carbendazim and benomyl fungicides, organochlorine pesticides, pyrethroids, organophosphates, and even the relatively new neonicotinoid pesticides have also been found to contaminate food [28,62]. Therefore, increased toxicity might be triggered by the simultaneous presence of multiple pesticides, which underscores the necessity for comprehensive risk assessments of pesticide mixtures [63,64].

Reproductive and developmental toxicity has been set as a threshold phenomenon to develop the reference exposure dose of carbendazim by multiple countries and organizations. The Joint FAO/WHO Meeting on Pesticide Residues (JMPR) adopted 0.1 mg kg^−1^ day^−1^ as the ARfD for child-bearing women based on a study of developmental toxicity in rabbits [65], and the Australian Pesticides and Veterinary Medicines Authority set the ARfD at 0.05 mg kg^−1^ day^−1^ based on a testicular toxicity study in rats [66]. Here, we implemented the most conservative ADI and ARfD of 0.02 mg kg^−1^ day^−1^ for both as developed by the EFSA and adopted in the EU from developmental data in rats and rabbits (NOAEL of 10 mg kg^−1^ day^−1^) with a safety factor of 500 [67]. The exceedance of acute exposure risk found in this study suggested that potential developmental toxicity to a fetus may be induced if high-residue food is consumed by pregnant women. An exceedance of the ARfD has also been identified for tomatoes, apples, and pears in the EU, representing 336%, 174%, and 162% of the ARfD [4]. Therefore, it is imperative to implement rigorous oversight of carbendazim residues in agricultural products, particularly in vegetables. Additionally, a reassessment of MRLs is warranted. The highest residue detected in cereals in this study was 5.2 mg kg^−1^ in rice, leading to corresponding ARs of consuming cereals of up to 4.6 for children. Even if the highest residue in rice was reduced to the current MRL of 2 mg kg^−1^, the corresponding ARs could still be as high as 1.8 for children.

### 4.5 Study strengths and limitations

The strengths of our study include the analysis of 66 different agricultural products (totaling 117,289 samples) that included 37 kinds of vegetables, 17 kinds of fruits, 9 kinds of mushrooms, 2 kinds of cereals, and tea all over China across the decade from 2011 to 2020. To our knowledge, this is one of the very few studies that has identified pesticide (carbendazim) residue in such a large scale of time and space and with such a wide range of foods. It's also the first study to provide an overall picture of the temporal and spatial variations of carbendazim in various foods across China. Furthermore, chronic and acute exposures and health risks were estimated using probabilistic assessments (Monte Carlo simulation), yielding more relevant information for Chinese consumers. Further analysis of pesticide usage and relevant national policy discussed the potential underlying reasons for spatial-temporal variation and laid out a means to understand and counteract the pesticide residue problem.

This study has several limitations, however. The sample sizes in the initial years of the study were comparatively small, particularly in 2011. Moreover, the analysis was restricted to carbendazim, which could be expanded in future research to include additional pesticides, thereby offering a more comprehensive characterization of pesticide usage in China and facilitating a more holistic estimation of combined health risks. Despite these limitations, the study illuminates the prevalence of carbendazim in a variety of agricultural commodities and ascertains the direct effects of policies to lower pesticide use on reducing residues in foods. Further follow-up studies to validate the findings in this study, particularly regarding other pesticides, appear warranted.

## 5 Conclusions

Fruits represent the primary source of carbendazim exposure for consumers in China, while vegetables are the food where consumers encounter high levels of carbendazim. The decrease in carbendazim from 2015 may have resulted from national policies, such as an action plan for zero growth of pesticide use by 2020. Still, high carbendazim found in several provinces usually included multiple categories of agricultural products, suggesting that comprehensive control is needed in these provinces, not just for carbendazim but also for other pesticides. The spatial-temporal variations of carbendazim observed in this study demonstrated that carbendazim is a representative substance that reflects broader pesticide usage trends in China. The exceedance of acute exposure risk derived from probabilistic exposure simulations further revealed carbendazim abuse and contamination problems in agricultural products in China and pointed out the derived health hazards to the population, such as developmental toxicity. Effective policies, including the redefinition of the maximum residue limits, need to be developed and implemented immediately to regulate and limit carbendazim use and food contamination even further, and these could also help to control other pesticides.

## CRediT authorship contribution statement

**Dou Wang**: Conceptualization, Methodology, Formal Analysis, Investigation, Data Curation, Writing - Original Draft. **Guiling Yang**: Resources, Conceptualization, Formal Analysis, Validation, Supervision, Writing - Review & Editing. **Xiao Yun**: Visualization, Software, Methodology, Investigation, Data Curation. **Ting Luo, Hao Guo**: Investigation, Data Curation, Software, Visualization. **Liying Pan**: Investigation, Data Curation, Software. **Wei Du**: Data Curation, Methodology, Validation. **Yanhua Wang**: Investigation, Data Curation, Software. **Qiang Wang**: Resources, Project Administration, Supervision. **Pu Wang, Qinghua Zhang**: Data Curation, Validation. **Yun Li**: Resources, Project Administration, Formal Analysis, Funding Acquisition. **Nan Lin**: Software, Validation, Formal Analysis, Investigation, Data Curation, Visualization, Writing - Original Draft, Writing - Review & Editing.

## Declaration of competing interest

The authors declare that they have no known competing financial interests or personal relationships that could have influenced the work reported in this paper.
