# Supplementary material for: Carbendazim residue in plant-based foods in China: Consecutive surveys from 2011 to 2020
Source: Environ Sci Ecotechnol. 2023 Jul 11;17:100301. doi: 10.1016/j.ese.2023.100301 (PMC10407663; doi:10.1016/j.ese.2023.100301)
Supplement: Multimedia component 1 [file mmc1.docx]

**Supplementary Material**

**Carbendazim residue in plant-based foods in China:**

**Consecutive surveys from 2011 to 2020**

Dou Wang ^a,1^, Guiling Yang ^a,1^, Xiao Yun ^b^, Ting Luo ^a^, Hao Guo ^a^, Liying Pan ^a^, Wei Du ^c^, Yanhua Wang ^a^, Qiang Wang ^a^, Pu Wang ^d^, Qinghua Zhang ^e^, Yun Li ^f,*^, Nan Lin ^g,*^

*^a^ State Key Laboratory for Managing Biotic and Chemical Threats to the Quality and Safety of Agro-products, Laboratory (Hangzhou) for Risk Assessment of Agricultural Products of Ministry of Agriculture, Institute of Agro-product Safety and Nutrition, Zhejiang Academy of Agricultural Sciences, Hangzhou 310021, Zhejiang, China*

*^b^ College of Urban and Environmental Sciences, Peking University, Beijing, P. R. China 100871*

*^c^ Yunnan Provincial Key Laboratory of Soil Carbon Sequestration and Pollution Control, Faculty of Environmental Science & Engineering, Kunming University of Science &Technology, Kunming 650500, China.*

*^d^ Hubei Key Laboratory of Industrial Fume and Dust Pollution Control, School of Environment and Health, Jianghan University, Wuhan, 430056, China*

*^e^ State Key Laboratory of Environmental Chemistry and Ecotoxicology, Research Center for Eco-Environmental Sciences, Chinese Academy of Sciences, Beijing 100085, China*

*^f^ Key Laboratory of Agro-Product Quality and Safety of Ministry of Agriculture, Institute of Quality Standards and Testing Technology for Agro-Products, Chinese Academy of Agricultural Sciences, Beijing, P. R. China 100081*

*^g^ Department of Environmental Health, School of Public Health, Shanghai Jiao Tong University, Shanghai, P. R. China 200025*

^1^ These authors contributed equally.

* Corresponding Authors:

**Nan Lin, Ph.D.**

Associate Professor, Department of Environmental Health

School of Public Health, Shanghai Jiao Tong University

280 South Chongqing Rd, Shanghai, P. R. China 200025

Tel: +86-18811461625 Email: [linnan5945@sjtu.edu.cn](mailto:linnan5945@sjtu.edu.cn)

**Yun Li, Ph.D.**

Institute of Quality Standards and Testing Technology for Agro-Products

Chinese Academy of Agricultural Sciences

NO.12 Zhong-guan-cun South Street, Beijing, P. R. China 100081

Tel: +86-18610001890 Email: [gz-liyun@126.com](mailto:gz-liyun@126.com)

## Exposure and risk assessment

For chronic risks, exposures were assessed based on the combined consumption of four categories of foods in provided years, then averaged by the weight of exposure duration (years) to the whole years of the population, see Eq. 1.

$\mathrm{EXP}_{Ch}=\sum_{j} \left[ \frac{\sum_{i}^{n=4} \left( C_{i}\times{DI}_{i} \right)}{{bw}_{j}}\times\frac{{ED}_{j}}{Y} \right]$ (1)

where, *EXP_Ch_* is the estimated average daily carbendazim exposure (mg kg^-1^ day^-1^) in diet during the exposure duration in the corresponding population, *C_i_* is the residue concentration (mg kg^-1^) in each category (i) of food measured in this study, *DI_i_* is the daily consumption of the food (g day^-1^), *bw_j_* is the body weight (kg) in the corresponding age (j) of the population, *ED_j_* is the exposure duration (year), *Y* is the years of the corresponding population.

Acute risks were assessed according to carbendazim exposure in each category of food; weighted-average body weight and daily food consumption were in prior obtained also based on the ratio of exposure years to the whole years of the population, see Eq. 2.

$\mathrm{EXP}_{Ac\_Food}=\frac{C\times\sum\left( {DI}_{j}\times\frac{{ED}_{j}}{Y} \right)}{\sum\left( {bw}_{j}\times\frac{{ED}_{j}}{Y} \right)}$ (2)

where, *EXP_Ac_Food_* is the estimated dietary carbendazim exposure (mg kg^-1^ day^-1^) in a day in certain category of food (vegetables, fruits, cereals or potatoes) among the corresponding population, *C* is the residue concentration (mg kg^-1^) in the certain food measured in this study, *DI_j_* is the daily consumption of the food (g day^-1^) in the corresponding age (j) of the population, *bw_j_* is the body weight (kg), *ED_j_* is the exposure duration (year), *Y* is the years of the corresponding population.

Both chronic risk (CR) of carbendazim exposure from combined daily consumption of four categories of food, and acute risk (AR) from certain category of food in a day in population were further estimated by the ratio of the up-calculated exposure to the European Food Safety Authority (EFSA) recommended acceptable daily intake (ADI, for chronic risk) and acute reference dose (ARfD, for acute risk) of both 0.02 mg kg^-1^ day^-1^ (Eqs. 3 and 4).

$CR=\frac{\mathrm{EXP}_{Ch}}{ADI}$ (3)

$AR=\frac{\mathrm{EXP}_{Ac\_Food}}{ARfD}$ (4)

Table S1. Food information, carbendazim limits of detection (LOD), and maximum residue levels (MRLs) of 66 kinds of foods.

| **Category** | **Sub-category** | **Name** | **Scientific name** | **LOD^*^ (mg kg^-1^)** | **MRL (mg kg^-1^)** | | | | |
| --- | --- | --- | --- | --- | --- | --- | --- | --- | --- |
|  |  |  |  |  | **2012** | **2014** | **2016** | **2019** | **2021** |
| **Vegetable** | Fruit vegetables | Tomato | *Lycopersicon esculentum* | 0.00012 | 3 | 3 | 3 | 3 | 3 |
|  |  | Leguminous vegetables | */* | 0.00012 – 0.001 |  | 0.5 | 0.5 | 0.5 | 0.5 |
|  |  | Eggplant | *Solanum melongena L.* | 0.00012 – 0.001 |  |  |  | 3 | 3 |
|  |  | Balsam pear | *Momordica charantia L.* | 0.00012 – 0.001 |  |  |  |  | 2^†^ |
|  |  | Pumpkin | *Cucurbita moschata (Duch. ex Lam.) Duch. ex Poiret* | 0.00012 – 0.002 |  |  |  |  | 2^†^ |
|  |  | Summer squash | *Cucurbita pepo L.* | 0.00012 – 0.001 |  | 0.5 | 0.5 | 0.5 | 0.5 |
|  |  | Sponge gourd | *Luffa cylindrica (L.) Roem.* | 0.00012 – 0.001 |  |  |  |  | 2^†^ |
|  |  | Chinese wax gourd | *Benincasa hispida (Thunb.) Cogn.* | 0.00012 – 0.001 |  |  |  |  | 2^†^ |
|  |  | Cucumber | *Cucumis sativus L.* | 0.00012 – 0.001 | 0.5 | 0.5 | 0.5 | 2 | 2 |
|  |  | Okra | *Abelmoschus esculentus (L.) Moench* | 0.00012 |  |  |  |  | 0.5^†^ |
|  |  | Hot pepper | *Capsicum annuum L.* | 0.00012 – 0.001 | 2 | 2 | 2 | 2 | 2 |
|  | Leafy vegetables | Chinese Cabbage | *Brassica pekinensis (Lour.) Rupr.* | 0.00012 – 0.001 |  |  |  |  | 0.5^†^ |
|  |  | Spinach | *Spinacia oleracea L.* | 0.00012 – 0.001 |  |  |  |  | 0.5^†^ |
|  |  | Amaranth | *Amaranthus tricolor L.* | 0.00012 – 0.001 |  |  |  |  | 0.5^†^ |
|  |  | Bok choy | *Brassica rapa var. chinensis (Linnaeus) Kitamura* | 0.00012 – 0.001 |  |  |  |  | 0.5^†^ |
|  |  | Water spinach | *Ipomoea aquatica Forsk* | 0.00012 – 0.001 |  |  |  |  | 0.2^†^ |
|  |  | Chinese chive | *Allium tuberosum Rottler ex Sprengle* | 0.00012 – 0.001 | 2 | 2 | 2 | 2 | 2 |
|  |  | Cabbage flowering stalk | *Brassica campestris L. ssp.chinensis var.utilis Tsen et* | 0.00012 |  |  |  |  | 0.5^†^ |
|  |  | Coriander leaf | *Coriandrum sativum L.* | 0.00012 – 0.001 |  |  |  |  | 0.5^†^ |
|  |  | Endive lettuce | *Lactuca sativa var. ramose Hort.* | 0.00012 – 0.001 |  |  |  |  | 5^†^ |
|  |  | Romaine lettuce | *Lactuca sativa var longifoliaf. Lam* | 0.00012 – 0.001 |  |  |  |  | 5^†^ |
|  |  | Vinespinach | *Basella alba L.* | 0.00012 |  |  |  |  | 5^†^ |
|  |  | Celery stem | *Apium graveolens L.* | 0.00012 – 0.001 |  |  |  |  | 0.5^†^ |
|  |  | Cabbage | *Brassica oleracea L.var.capitata L.* | 0.00012 – 0.001 |  | 0.5 | 0.5 | 0.5 | 0.5 |
|  |  | Common spiderflower | *Brassica oleracea var. botrytis Linnaeus* | 0.00012 – 0.001 |  |  |  |  | 0.5^†^ |
|  |  | Leaf mustard | *Brassica juncea (L.) Czern. et Coss.* | 0.00012 |  |  |  |  | 0.5^†^ |
|  |  | Crown daisy | *Glebionis coronaria (Linnaeus) Cassini ex Spach* | 0.00012 – 0.001 |  |  |  |  | 5^†^ |
|  | Stalk and stem | Yam | *Dioscorea polystachya Turczaninow* | 0.00012 |  |  |  |  | 0.2 |
|  | vegetables | Potato | *Solanum tuberosum L.* | 0.00012 – 0.002 |  |  |  |  | 0.2^†^ |
|  |  | Lotus root | *Nelumbo nucifera Gaertn* | 0.00012 – 0.01 |  |  |  | 0.2 | 0.2 |
|  |  | Radish & Carrot^‡^ | *Raphanus sativus L.* & *Daucus carota subsp. sativus* | 0.00012 |  | 0.2^§^ | 0.2^§^ | 0.2^§^ | 0.2^§^ |
|  |  | Lettuce stem | *Lactuca sativa L.* | 0.00012 |  | 5 | 5 |  | 5 |
|  |  | Manchurian wild rice | *Zizania latifolia (Griseb.) Stapf* | 0.00012 – 0.001 |  |  |  |  | 0.2^†^ |
|  |  | Water chestnut | *Eleocharis dulcis (Burm. f.) Trin.* | 0.00012 – 0.01 |  |  |  |  |  |
|  |  | Arrowhead | *Sagittaria trifolia L. var. sinensis (Sims.) Makino* | 0.00012 – 0.01 |  |  |  |  |  |
|  |  | Asparagus | *Asparagus officinalis L.* | 0.00012 – 0.001 | 0.1 | 0.1 | 0.5 | 0.5 | 0.5 |
|  |  | Allium vegetables | */* | 0.00012 – 0.001 |  |  |  |  | 2^†^ |
| **Fruit** | Melons | Watermelon | *Citrullus lanatus (Thunb.) Matsum. et Nakai* | 0.0001 – 0.00012 | 0.5 | 0.5 | 2 | 2 | 2 |
|  |  | Casaba | *Cucumis melo L.* | 0.0001 – 0.008 |  |  |  |  | / |
|  | Pome and stone fruits | Apple | *Malus pumila Mill.* | 0.00012 – 0.001 | 3 | 3 | 5 | 5 | 5 |
|  |  | Pear | *Pyrus spp* | 0.0001 – 0.0005 | 3 | 3 | 3 | 3 | 3 |
|  |  | Plum | *Prunus salicina Lindl.* | 0.0001 – 0.00012 | 0.5 | 0.5 | 0.5 | 0.5 | 0.5 |
|  |  | Peach | *Amygdalus persica L.* | 0.0001 – 0.0005 | 2 | 2 | 2 | 2 | 2 |
|  |  | Chinese date | *Ziziphus jujube Mill.* | 0.00012 – 0.0005 | 0.5 | 0.5 | 0.5 | 0.5 | 0.5 |
|  |  | Pomegranate | *Punica granatum L.* | 0.00012 |  |  |  | 1 | 0.5^†^ |
|  |  | False sour cherry | *Cerasus pseudocerasus (Lindl.) G. Don* | 0.00012 – 0.001 | 0.5 | 0.5 | 0.5 | 0.5 | 0.5 |
|  |  | Bayberry | *Myrica rubra (Lour.) S. et Zucc.* | 0.0001 – 0.07 |  |  |  | 1 | 1 |
|  |  | Loquat | *Eriobotrya japonica (Thunb.) Lindl.* | 0.0001 – 0.0052 |  | 3 | 3 | 3 | 3 |
|  |  | Lychee | *Litchi chinensis Sonn.* | 0.00012 | 0.5 | 0.5 | 0.5 | 0.5 | 0.5 |
|  |  | Banana | *Musa nana Lour.* | 0.0001 – 0.064 | 0.1 | 0.1 | 2 | 2 | 2 |
|  | Citrus | Citrus | *Citrus L.* | 0.0001 – 0.01 | 5 & 0.5 | 5 & 0.5 | 5 & 0.5 | 5 | 5 |
|  | Berries | Grape | *Vitis vinifera L.* | 0.0001 – 0.00012 | 3 | 3 | 3 | 3 | 3 |
|  |  | Strawberry | *Fragaria × ananassa Duch.* | 0.00012 – 0.001 | 0.5 | 0.5 | 0.5 | 0.5 | 0.5 |
|  |  | Chinese kiwi fruit | *Actinidia chinensis Planch* | 0.0001 – 0.031 | 0.5 | 0.5 | 0.5 | 0.5 | 5 |
| **Mushroom** |  | Shitake mushroom | *Lentinus edodes (Berk.) Sing* | 0.001 |  |  |  |  | 3^†^ |
|  |  | Button mushroom | *Agaricus bisporus (Large) Sing.* | 0.001 |  |  |  |  | 3^†^ |
|  |  | Oyster mushroom | *Pleurotus ostreatus* | 0.001 |  |  |  |  | 3^†^ |
|  |  | Columnar agroc | *Agrocybe aegerita* | 0.001 |  |  |  |  | 3^†^ |
|  |  | Gold needle mushroom | *Flammulina velutipes* | 0.001 |  |  |  |  | 3^†^ |
|  |  | King oyster mushroom | *Pleurotus eryngii* | 0.001 |  |  |  |  | 3^†^ |
|  |  | Straw mushroom | *Volvariella volvacea (Bull.) Singer* | 0.001 |  |  |  |  | 3^†^ |
|  |  | Wood ear fungus | *Auricularia auricula (L.ex Hook.)Underwood* | 0.001 |  |  |  |  | 3^†^ |
|  |  | Silver ear fungus | *Tremella fuciformis Berk.* | 0.001 |  |  |  |  | 3^†^ |
| **Cereal** |  | Rice | *Oryza sativa L.* | 0.005 | 2 | 2 | 2 | 2 | 2 |
|  |  | Wheat | *Triticum aestivum L.* | 0.001 | 0.05 | 0.05 | 0.5 | 0.5 | 0.5 |
| **Tea** |  | Tea | */* | 0.001 | 5 | 5 | 5 | 5 | 5 |

^*^LOD differed in different years for the same food.

^†^MRLs are from guidelines of the Ministry of Agriculture and Rural Affairs of the People's Republic of China.

^‡^Radishes and carrots were mixed when recording data.

^§^MDL (0.2 mg kg^-1^) is for carrot (Radish has no MDL).

Table S2. Parameters of exposure assessments.

| **Target population** | **Age** | **Gender** | **Body weight (kg)**^*^ | **Daily consumption (g)** | | | |
| --- | --- | --- | --- | --- | --- | --- | --- |
|  |  |  |  | **Vegetables** | **Fruits** | **Cereals** | **Potatoes** |
| Children | 2 – 7 | Both | 16.6 | 194.8 | 65.8 | 218.3 | 24.3 |
| Adolescent (Both) | 8 – 12 | Both | 31.6 | 272.4 | 101.0 | 336.0 | 44.7 |
| Adolescent Male | 13 – 19 | Male | 53.4 | 396.7 | 122.5 | 461.8 | 49.4 |
| Adolescent Female | 13 – 19 | Female | 48.5 | 317.9 | 110.8 | 368.8 | 48.7 |
| Adult Male | 20 – 50 | Male | 65.3 | 436.4 | 78.0 | 475.7 | 56.4 |
| Adult Female | 20 – 50 | Female | 55.6 | 412.1 | 107.0 | 386.9 | 52.9 |
| Adult Male | 51 – 65 | Male | 66.0 | 477.9 | 80.2 | 461.0 | 55.1 |
| Adult Female | 51 – 65 | Female | 59.5 | 447.0 | 86.2 | 391.8 | 50.3 |
| Adult Male | > 65 | Male | 62.4 | 413.3 | 64.4 | 398.8 | 41.7 |
| Adult Female | > 65 | Female | 56.6 | 364.1 | 64.7 | 326.3 | 36.6 |

^*^ All other data except for body weight were from the Fifth China Total Diet Study and calculated as mean values. Body weight was obtained and calculated from the Exposure Factors Handbook of Chinese Population regarding the corresponding population with the close age as the Fifth China Total Diet Study [1].

Table S3. Chronic exposure to carbendazim from the combined intake of four categories of foods (vegetables, fruits, cereals, and potatoes), and acute exposure from each category of food in five populations.

| **Population** | **Food** | **Daily Exposure (mg kg^-1^ day^-1^)** | | | | |
| --- | --- | --- | --- | --- | --- | --- |
|  |  | **Min** | **25%** | **Median** | **75%** | **Max** |
| **Chronic exposure** |  |  |  |  |  |  |
| Children | Combined | 2.14 × 10^-6^ | 2.95 × 10^-5^ | 4.16 × 10^-5^ | 2.23 × 10^-4^ | 0.27 |
| Adolescent Male | Combined | 1.58 × 10^-6^ | 2.21 × 10^-5^ | 2.86 × 10^-5^ | 1.55 × 10^-4^ | 0.20 |
| Adolescent Female | Combined | 1.42 × 10^-6^ | 2.08 × 10^-5^ | 2.69 × 10^-5^ | 1.51 × 10^-4^ | 0.20 |
| Adult Male | Combined | 1.13 × 10^-6^ | 1.64 × 10^-5^ | 2.04 × 10^-5^ | 9.07 × 10^-5^ | 0.14 |
| Adult Female | Combined | 1.17 × 10^-6^ | 1.56 × 10^-5^ | 1.96 × 10^-5^ | 1.04 × 10^-4^ | 0.18 |
| **Acute exposure** |  |  |  |  |  |  |
| Children | Vegetable | 4.24 × 10^-7^ | 6.51 × 10^-7^ | 7.25 × 10^-7^ | 8.21 × 10^-7^ | 0.24 |
|  | Fruit | 1.23 × 10^-7^ | 2.18 × 10^-7^ | 2.54 × 10^-7^ | 4.58 × 10^-5^ | 0.01 |
|  | Cereal | 1.11 × 10^-6^ | 7.40 × 10^-6^ | 3.08 × 10^-5^ | 3.69 × 10^-5^ | 0.09 |
|  | Potato | 5.34 × 10^-8^ | 8.07 × 10^-8^ | 8.89 × 10^-8^ | 9.90 × 10^-8^ | 0.01 |
| Adolescent Male | Vegetable | 2.80 × 10^-7^ | 4.32 × 10^-7^ | 4.80 × 10^-7^ | 5.46 × 10^-7^ | 0.19 |
|  | Fruit | 7.21 × 10^-8^ | 1.40 × 10^-7^ | 1.64 × 10^-7^ | 3.01 × 10^-5^ | 0.01 |
|  | Cereal | 7.20 × 10^-7^ | 5.20 × 10^-6^ | 2.16 × 10^-5^ | 2.60 × 10^-5^ | 0.07 |
|  | Potato | 3.90 × 10^-8^ | 5.87 × 10^-8^ | 6.49 × 10^-8^ | 7.25 × 10^-8^ | 0.01 |
| Adolescent Female | Vegetable | 2.48 × 10^-7^ | 4.00 × 10^-7^ | 4.45 × 10^-7^ | 5.07 × 10^-7^ | 0.17 |
|  | Fruit | 7.98 × 10^-8^ | 1.41 × 10^-7^ | 1.65 × 10^-7^ | 3.02 × 10^-5^ | 0.01 |
|  | Cereal | 7.58 × 10^-7^ | 4.82 × 10^-6^ | 2.01 × 10^-5^ | 2.41 × 10^-5^ | 0.06 |
|  | Potato | 3.86 × 10^-8^ | 6.24 × 10^-8^ | 6.91 × 10^-8^ | 7.68 × 10^-8^ | 0.01 |
| Adult Male | Vegetable | 2.41 × 10^-7^ | 3.76 × 10^-7^ | 4.19 × 10^-7^ | 4.75 × 10^-7^ | 0.16 |
|  | Fruit | 3.53 × 10^-8^ | 6.32 × 10^-8^ | 7.44 × 10^-8^ | 1.35 × 10^-5^ | 0.00 |
|  | Cereal | 5.73 × 10^-7^ | 3.88 × 10^-6^ | 1.64 × 10^-5^ | 1.96 × 10^-5^ | 0.04 |
|  | Potato | 2.77 × 10^-8^ | 4.44 × 10^-8^ | 4.91 × 10^-8^ | 5.47 × 10^-8^ | 0.01 |
| Adult Female | Vegetable | 2.40 × 10^-7^ | 3.99 × 10^-7^ | 4.44 × 10^-7^ | 5.04 × 10^-7^ | 0.14 |
|  | Fruit | 4.79 × 10^-8^ | 8.85 × 10^-8^ | 1.03 × 10^-7^ | 1.93 × 10^-5^ | 0.01 |
|  | Cereal | 5.88 × 10^-7^ | 3.70 × 10^-6^ | 1.54 × 10^-5^ | 1.85 × 10^-5^ | 0.04 |
|  | Potato | 2.80 × 10^-8^ | 4.67 × 10^-8^ | 5.17 × 10^-8^ | 5.75 × 10^-8^ | 0.01 |

Table S4. Carbendazim residue in foods in previous studies and the present study.

| **Country** | **Time** | **Food (Sample numbers)** | **LOD or LOQ (mg kg^-1^)** | **DF** | **Carbendazim (mg kg^-1^)** | | **Ref.** |
| --- | --- | --- | --- | --- | --- | --- | --- |
|  |  |  |  |  | **Mean** | **Range** |  |
| **Vegetable** |  |  |  |  |  |  |  |
| Karachi, Pakistan | 2000 – 2003 | Vegetable (320) | / | 3.1% | / | / | [2] |
| Pakistan | 2003 | Tomato (/)  Potato (/) | /  / | /  / | 0.21  0.13 | /  / | [3] |
| Aegean, Turkey | 2010 – 2012 | Vegetable (850) | / | 4.5% | / | ND – 1.2 | [4] |
| Bogota, Colombia | 2011 | Tomato (400) | 0.01 | 19.5% | 0.05 | ND – 0.74 | [5] |
| China | 2014 – 2016 | Vegetable (20496) | 0.01 | 8.1% | 0.23 | ND – 34.8 | [6] |
| Rupandehi, Nepal | 2017 | Vegetable (86) | 0.001 | 29.0% | 0.02, 0.05, 0.02  (Eggplant, Tomato, Chilli) | ND – 0.34 | [7] |
| Meru County, Kenya | 2020 | Vegetable (66) | 0.0001 | 65.2% | 0.005 (Tomato)  0.002 (French beans)  ND (Kale) | 0.0003 – 0.05 (Tomato)  0.0002 – 0.01 (French beans)  ND (Kale) | [8] |
| Cameroon | 2017 | Cowpea, chili pepper and other dry foods (72) | 0.0001/0.0004 | 1.4% | 0.0014 | 0.0014 | [9] |
| Cameroon | 2017 | Cowpea, chili pepper and other dry foods (160) | 0.0001/0.0004 | 8.1% | 0.0623 | 0.0021 – 0.4090 | [10] |
| China | 2011 – 2020 | Vegetable (74029) | 0.0001 – 0.01 | 7.7%^*^ | 0.026 | ND – 110 | This study |
| **Fruit** |  |  |  |  |  |  |  |
| Valencia, Spain | 2002 | Peach and nectarine (159) | 0.02 | 34% | 0.25 (for 34% samples) | ND – 0.9 | [11] |
| Pakistan | 2003 | Banana (/) | / | / | 0.067 | / | [3] |
|  |  | Plum (/) | / | / | 0.058 | / |  |
| Aegean, Turkey | 2010 – 2012 | Fruit (573) |  | 6.3% | / | ND – 0.63 | [4] |
| China | 2013 – 2015 | Peach (312) | 0.0004 | 60.6% | 0.17 | ND – 3.4 | [12] |
| China | 2011 – 2020 | Fruit (24607) | 0.0001 – 0.07 | 26.4%^*^ | 0.029 | ND – 8.4 | This study |
| **Mushroom** |  |  |  |  |  |  |  |
| Aegean, Turkey | 2010 – 2012 | Mushroom (58) | / | 87.9% | / | ND – 3.53 | [4] |
| China | 2011 – 2020 | Mushroom (17275) | 0.001 | 11.3%^*^ | 0.023 | ND – 35.4 | This study |
| **Tea** |  |  |  |  |  |  |  |
| Hangzhou, China | 2018 | Tea (45) | 0.01 | 57.8% | / | ND – 0.36 | [13] |
| China | 2011 – 2020 | Tea (470) | 0.001 | 0.9%^*^ | 0.0007 | ND – 0.04 |  |
| **Total** |  |  |  |  |  |  |  |
| USA | 2017 | Total Diet Study (1064) | 0.01 | 7.0% | 0.0013 | ND – 0.04 | [14] |
|  | 2017 | All foods (6069) | 0.01 | 4.9% | / | / |  |
|  | 2018 | All foods (4404) | 0.01 | 4.8% | / | / |  |
|  | 2019 | All foods (4327) | 0.01 | 4.2% | / | / |  |
| China | 2011 – 2020 | Total (117289) | 0.0001 – 0.07 | 12.2%^*^ | 0.026 | ND – 110 | This study |

*DF of residue ≥ 0.01 mg kg^-1^.

Fig S1. The map showing samples distribution among target 31 provinces and their belonging geographical regions in mainland China.


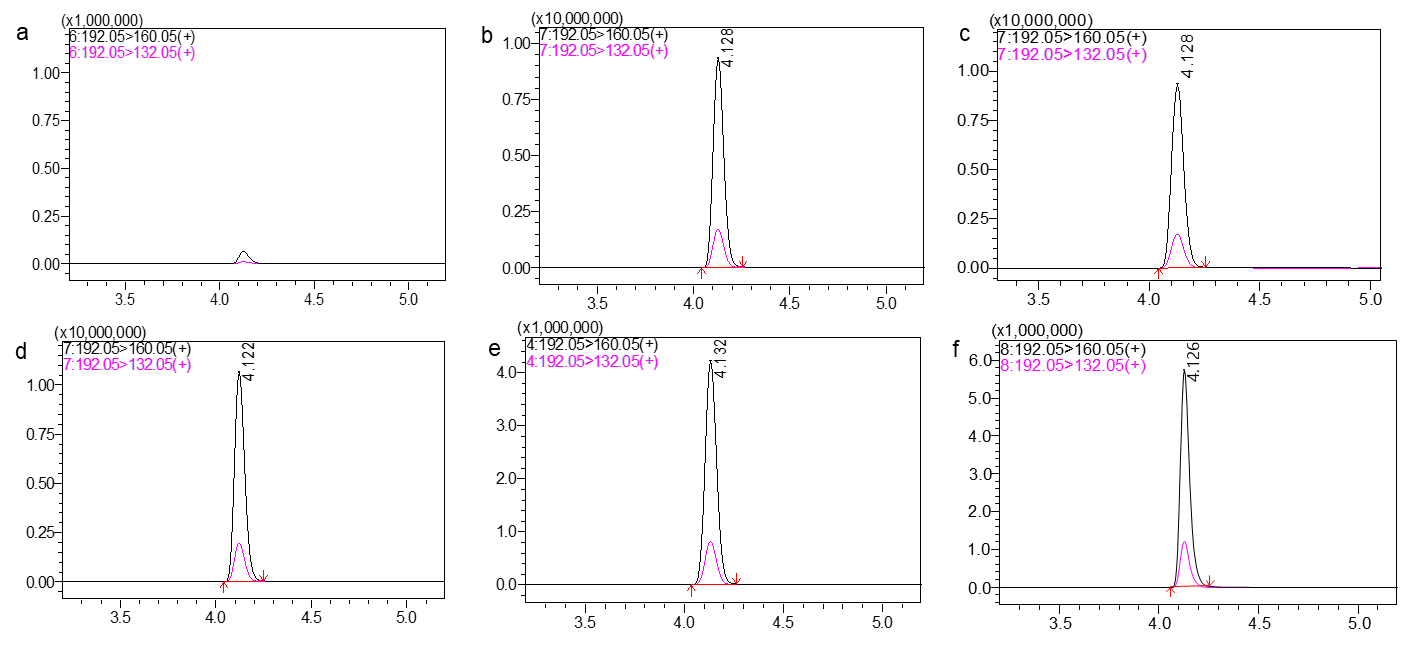


Fig S2. Chromatograms of carbendazim in a) blank celery stem matrix, b) carbendazim standard fortified celery stem matrix at 0.10 mg L^-1^, c) celery stem sample at 0.10 mg kg^-1^, d) cucumber sample at 0.10 mg kg^-1^, e) bayberry sample at 0.12 mg kg^-1^, and f) apple sample at 0.15 mg kg^-1^.


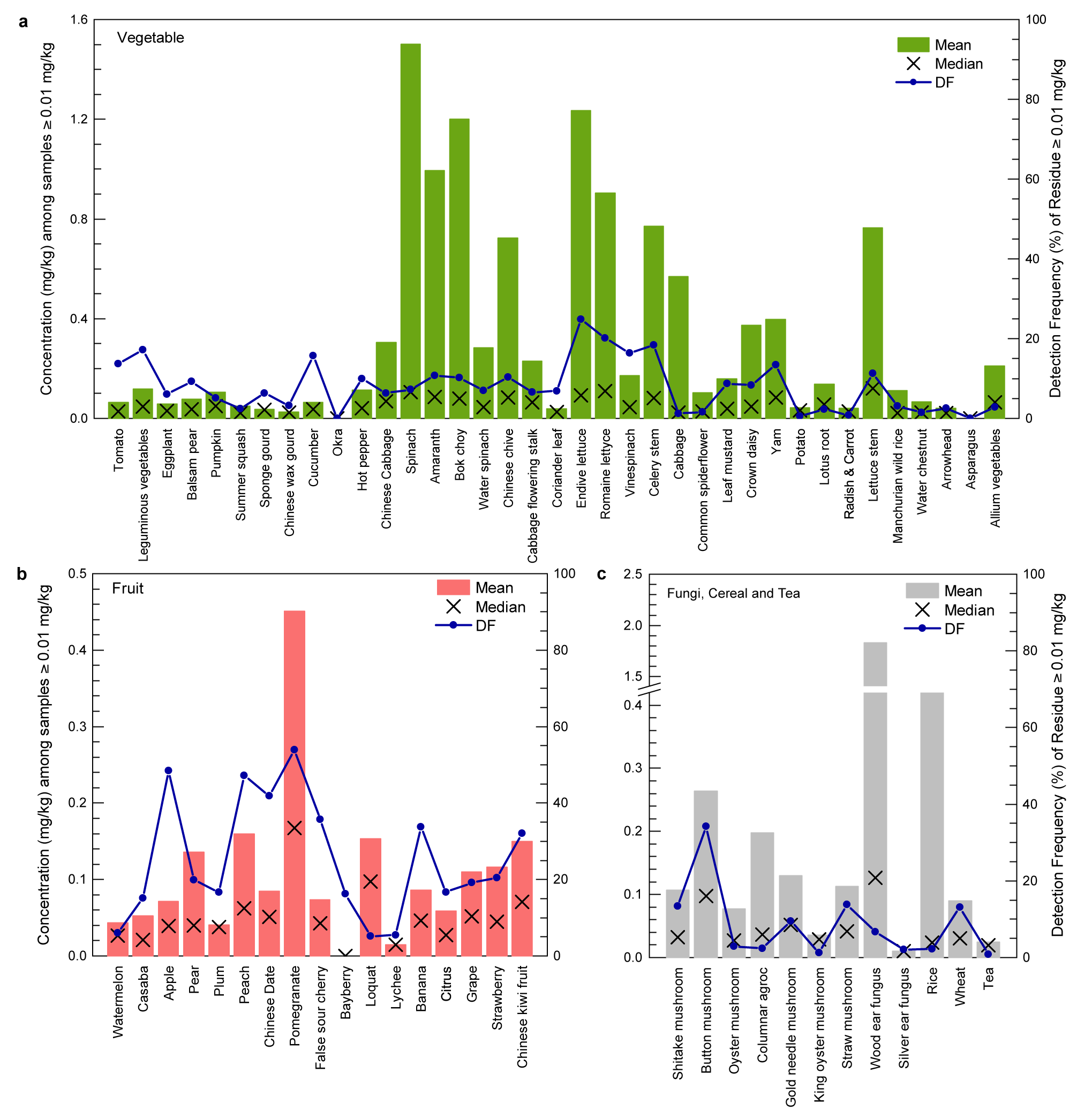


Fig S3. Detection frequency with carbendazim over 0.01 mg kg^-1^, median and mean concentrations among samples with carbendazim over 0.01 mg kg^-1^ in **a**, 37 vegetables, **b**, 17 fruits, and **c**, 9 mushrooms, 2 cereals and 1 tea.


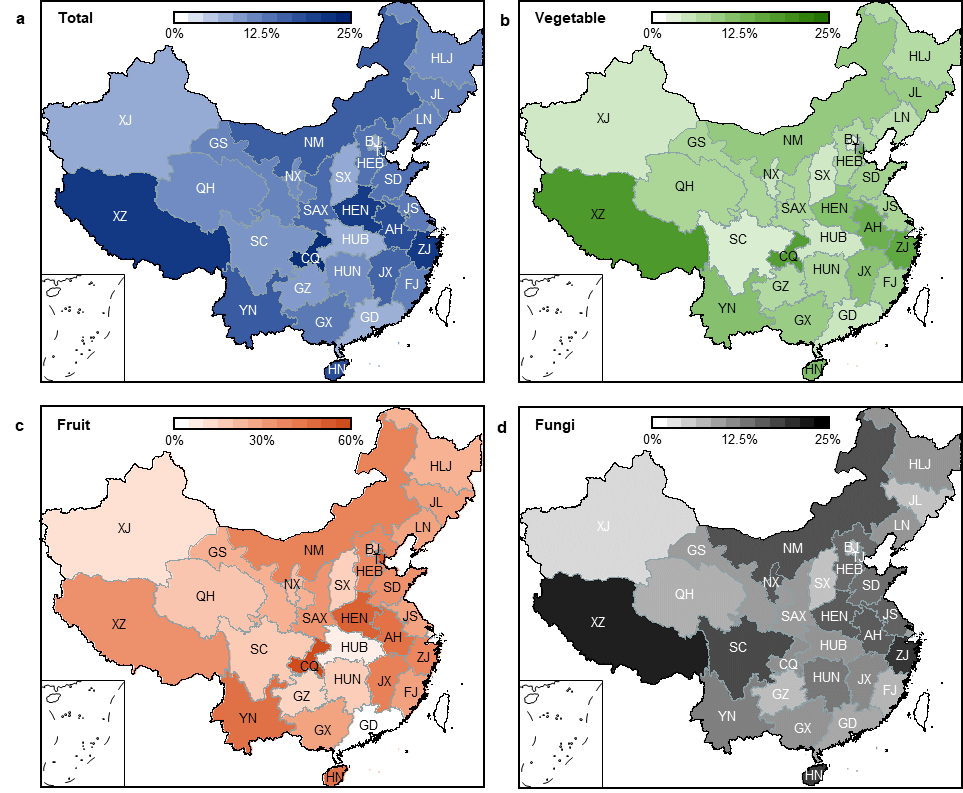


Fig S4. Spatial distribution of detection frequency of carbendazim over 0.01 mg kg^-1^ in **a**, total food samples, **b**, vegetables, **c**, fruits, and **d**, mushroom by province in mainland China during the decade of 2011–2020.


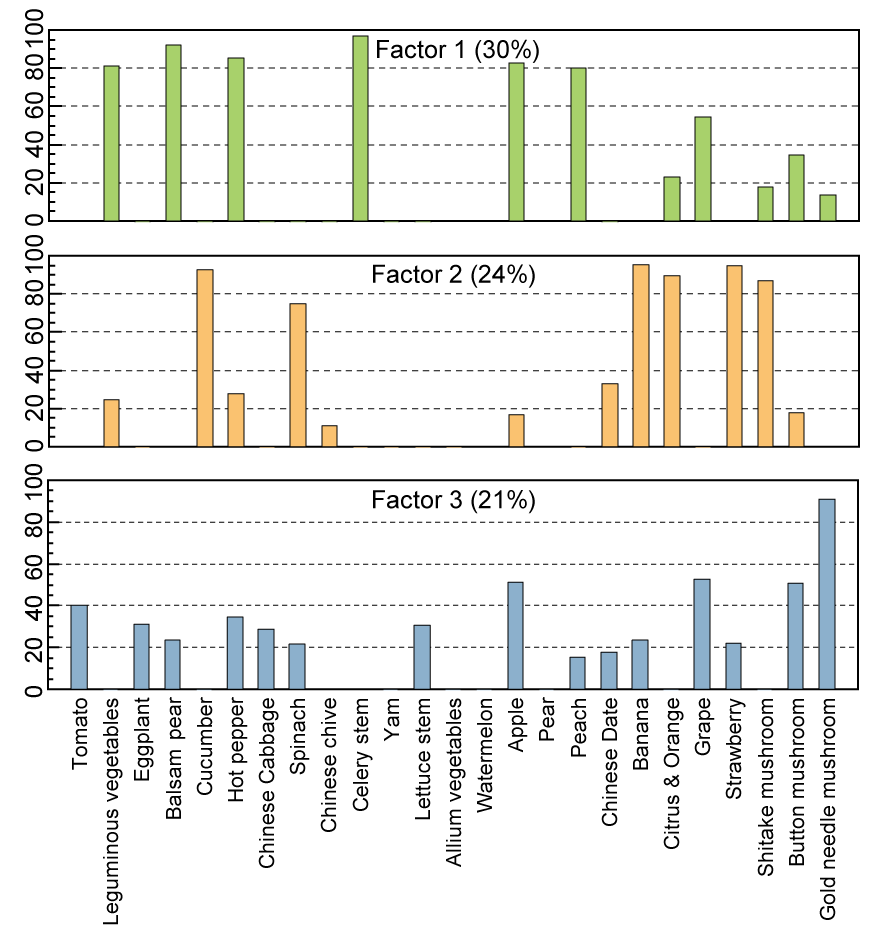


Fig S5. Factor loadings (source profiles) from PCA for samples with residue over 0.01 mg kg^-1^.

**Reference**

1. Ministry of Environmental Protection of China, Exposure Factors Handbook of Chinese Population, China Environmental Science Press: Beijing, China, 2013.
2. Z. Parveen, M.I. Khuhro, N. Rafiq, Monitoring of pesticide residues in vegetables (2000-2003) in Karachi, Pakistan, Bul. Environ. Contam. Toxicol. 74 (1) (2005) 170-176.
3. J.H. Syed, A. Alamdar, A. Mohammad, K. Ahad, Z. Shabir, H. Ahmed, S.M. Ali, G.A.S. Sani Syed, H. Bokhari, K.D.Gallagher et al, Pesticide residues in fruits and vegetables from Pakistan: a review of the occurrence and associated human health risks, Environ. Sci. Pollut. Res. 21 (23) (2014) 13367-13393.
4. G.T. Bakirci, D.B.Y. Acay, F. Bakirci, S. Otles, Pesticide residues in fruits and vegetables from the Aegean region, Turkey, Food Chem. 160 (2014) 379-392.
5. L.A. Arias, C.R. Bojaca, D.A. Ahumada, E. Schrevens, Monitoring of pesticide residues in tomato marketed in Bogota, Colombia, Food Control 35(1) (2014) 213-217.
6. X.M. Xu, J.Y. Chen, B.R. Li, L.J. Tang, Carbendazim residues in vegetables in China between 2014 and 2016 and a chronic carbendazim exposure risk assessment, Food Control 91 (2018) 20-25.
7. G. Bhandari, P. Zomer, K. Atreya, H.G.J. Mol, X.M. Yang, V. Geissen, Pesticide residues in Nepalese vegetables and potential health risks, Environ. Res. 172 (2019) 511-521.
8. G.M. Marete, V.O. Shikuku, J.O. Lalah, J. Mputhia, V.W. Wekesa, Occurrence of pesticides residues in French beans, tomatoes, and kale in Kenya, and their human health risk indicators, Environ. Monit. Assess. 192 (11) (2020) 692.
9. J.H.Y. Galani, M. Houbraken, A. Wumbei, J.F. Djeugap, D. Fotio, P. Spanoghe, Evaluation of 99 pesticide residues in major agricultural products from the western highlands zone of Cameroon using QuEChERS method extraction and LC-MS/MS and GC-ECD analyses, Foods 7(11) (2018) 184.
10. J.H.Y. Galani, M. Houbraken, A. Wumbei, J.F. Djeugap, D. Fotio, Y.Y. Gong, P. Spanoghe, Monitoring and dietary risk assessment of 81 pesticide residues in 11 local agricultural products from the 3 largest cities of Cameroon, Food Control 118 (2020) 107416.
11. C. Blasco, M. Fernandez, Y. Pico, G. Font, J. Manes, Simultaneous determination of imidacloprid, carbendazim, methiocarb and hexythiazox in peaches and nectarines by liquid chromatography-mass spectrometry, Anal. Chim. Acta. 461(1) (2002) 109-116.
12. Z.X. Li, J.Y. Nie, Z. Yan, Y. Cheng, F. Lan, Y.N. Huang, Q.S. Chen, X.B. Zhao, A. Li, A monitoring survey and dietary risk assessment for pesticide residues on peaches in China, Regul. Toxicol. Pharm. 97 (2018) 152-162.
13. Z. Li, Y.P. Jiang, Q. Lin, X. Wang, X.Z. Zhang, J. Xu, Z.M. Chen, Residue transfer and risk assessment of carbendazim in tea, J. Sci. Food Agriculure. 98(14) (2018) 5329-5334.
14. U.S. Food and Drug Administration, Pesticide Residue Monitoring Program Reports and Data, Available online: https://www.fda.gov/food/pesticides/pesticide-residue-monitoring-program-reports-and-data.
